# Isoliquiritin Apioside Suppresses *in vitro* Invasiveness and Angiogenesis of Cancer Cells and Endothelial Cells

**DOI:** 10.3389/fphar.2018.01455

**Published:** 2018-12-10

**Authors:** Aeyung Kim, Jin Yeul Ma

**Affiliations:** Korean Medicine (KM) Application Center, Korea Institute of Oriental Medicine (KIOM), Daegu, South Korea

**Keywords:** cancer, metastasis, angiogenesis, HT1080, isoliquiritin apioside, HIF-1α, NF-κB, MAPK

## Abstract

Several components isolated from *Glycyrrhizae radix rhizome* (GR), including glycyrrhizin, liquiritin, and liquiritigenin, have been shown to induce cancer cell death and inhibit cancer metastasis. Isoliquiritin apioside (ISLA), a component isolated from GR, has been effective for treating tetanic contraction and genotoxicity. However, the effects of ISLA on the metastasis and angiogenesis of malignant cancer cells and endothelial cells (ECs) have not been reported. In this study, we found that up to 100 μM ISLA did not affect cell proliferation but efficiently suppressed the metastatic ability of HT1080 cells, as assessed by scratch-wound migration, Transwell^®^ migration, scratch-wound invasion, Transwell^®^ invasion, and three-dimensional spheroid invasion. ISLA significantly decreased phorbol 12-myristate 13-acetate (PMA)-induced increases in matrix metalloproteinase (MMP) activities and suppressed PMA-induced activation of mitogen-activated protein kinase as well as NF-κB, which are involved in cancer metastasis. In addition, ILSA treatment reduced the production of pro-angiogenic factors in HT1080 cells, including MMP-9, placental growth factor, and vascular endothelial growth factor under normoxia as well as hypoxia conditions, by impairing the hypoxia-inducible factor-1α pathway. We also found that the abilities of human umbilical vein ECs to migrate across the Transwell^®^ and to form tube-like structures were significantly reduced by ISLA treatment. Moreover, using the chorioallantoic membrane assay, vessel formation with or without vascular endothelial growth factor was significantly suppressed by ISLA. These results suggested that ISLA possesses anti-metastatic and anti-angiogenic abilities in malignant cancer cells and ECs, with no cytotoxicity. ISLA may therefore be a safe and effective lead compound to develop anti-cancer drug for limiting the spread of primary tumors to distant organs to form secondary tumors.

## Introduction

Cancer metastasis, the dissemination of cancer cells from the location where they initially formed to anatomically distant parts of the body, is a hallmark of malignant tumors and a major cause of the high mortality of cancer patients (Leber and Efferth, 2009; Valastyan and Weinberg, 2011). Metastasis involves multiple highly coordinated steps, including loss of adhesions between cells and between cells and the extracellular matrix (ECM), invasion into the surrounding ECM, intravasation into the lumina of blood vessels, migration and extravasation to distant organs, and then colonization of metastatic foci at a secondary site (Patel et al., 2011; Alizadeh et al., 2014). Angiogenesis in the tumor mass is also required for invasive tumor growth and metastasis because newly formed vessels provide sufficient nutrients for tumor growth and are the principal route for the escape of tumor cells into the circulation and for the consequential development of distant metastatic foci (Rak et al., 1995; Folkman, 2002). Angiogenesis is initiated by pro-angiogenic factors and proteolytic enzymes released by tumor cells, which enhance migration of endothelial cells (ECs) toward tumors, proliferation of ECs, and the formation of functional vascular networks (Sridhar and Shepherd, 2003; Yadav et al., 2015). In many tumors, increased vascular density within the tumor mass was associated with metastases, poor prognoses and decreased survival times of cancer patients (Kaya et al., 2000; Offersen et al., 2003).

Among tumor-derived factors involved in metastasis and angiogenesis, matrix metalloproteinases (MMPs) are regarded as the most critical effectors. MMPs break down diverse substrates in the extracellular milieu for tumor invasion, modulate the bioavailability of growth factors for tumor proliferation, and promote tumor dissemination by degrading the vascular basement membrane and remodeling ECs (Kessenbrock et al., 2010). In addition, pro-angiogenic molecules secreted by tumor cells under hypoxic conditions, including vascular endothelial growth factor (VEGF), platelet-derived growth factor (PDGF), epidermal growth factor (EGF), and MMPs, can activate their receptors on the surface of ECs and trigger angiogenesis (Harris, 2002; Pugh and Ratcliffe, 2003; Vaupel, 2004). Suppression of metastasis and angiogenesis in tumors by targeting these factors and their upstream pathways is therefore considered to be a promising strategy for the control of malignant tumors.

*Glycyrrhizae* *radix et rhizoma* (GR), which is the root of *Glycyrrhiza uralensis*, has been used as a traditional Chinese medicine in clinical practice mainly to treat hepatitis, bowel diseases, cough, and pulmonary and skin diseases because of its ability to replenish the deficiencies in “Gi and Blood” (Jiang, 2005; Sekine-Osajima et al., 2009). Pharmacological studies have demonstrated antioxidant, anti-tumor, antitussive, anti-inflammatory, antiosteoporosis, anti-ulcerative colitis, detoxification, and neuroprotective activities of GR (Lee et al., 2012; Shi et al., 2014; Jeon et al., 2016; Rho et al., 2017). Several chemical components isolated from GR, including glycyrrhizin, liquiritigenin, and liquiritin, have been reported to possess anti-cancer activities that induce apoptosis and inhibit invasion (Huang et al., 2014; Wei et al., 2017; He et al., 2017; Meng and Lin, 2018). Another active component, isoliquiritin apioside (ISLA), showed marked modulatory activities against oxidative-stress-induced genotoxicity and significantly inhibited tetanic contractions of rat gastrocnemius muscles (Kaur et al., 2009; Lee et al., 2013). However, the effects of ISLA on the metastasis and angiogenesis of tumors have not been reported.

In the present study, we determined whether ISLA controls cancer cells and ECs by inhibiting metastasis and angiogenesis, using an *in vitro* and *in ovo* chick chorioallantoic membrane (CAM) assay. In addition, we investigated the underlying mechanisms of the anti-metastatic and anti-angiogenic activities of ISLA in detail.

## Materials and Methods

### Cell Culture

Human fibrosarcoma HT1080 cells were obtained from the Korean Cell Line Bank (KCLB, No. 10121) and maintained in RPMI1640 media (Hyclone Laboratories, Logan, UT, United States) with 10% fetal bovine serum (FBS, Hyclone Laboratories) and penicillin/streptomycin (Cellgro, Manassas, VA, United States) at 37°C in a humidified 5% CO_2_ incubator. Human umbilical vein endothelial cells (HUVECs) were obtained from Innopharmascreen (Asan, Republic of Korea), maintained in Endothelial Cell Growth Medium-2 (EGM-2, PromoCell, Heidelberg, Germany), and used for assays at passages 3–8.

### Chemicals and Antibodies

Isoliquiritin apioside (ISLA, ≥98% purity using high-pressure liquid chromatography, Catalog No. CFN90800, CAS No. 120926-46-7) was purchased from Faces Biochemical (Wuhan, China) and dissolved with 100% DMSO to 100 mM. Phorbol-12-myristate 13-acetate (PMA), mitomycin C from *Streptomyces caespitosus*, 4,6-diamidino-2-phenynolindole (DAPI), and cobalt(II) chloride (CoCl_2_) were purchased from Sigma Chemical Co. (St. Louis, MO, United States). Growth factor-reduced Matrigel basement membrane matrix and recombinant murine vascular endothelial growth factor165 (rMu VEGF165) were obtained from BD Biosciences (Bedford, MA, United States) and PromoKine (Heidelberg, Germany), respectively. Antibodies against MMP-9, MMP-2, p38, p-p38, ERK, p-ERK, JNK, p-JNK, Akt, p-Akt, mTOR, p-mTOR, p-4E-BP1, p-p70S6K, p65, TBP, and tubulin were obtained from Cell Signaling Technology (Danvers, MA, United States). Anti-HIF-1α antibody and horseradish peroxidase-conjugated anti-rabbit antibody were obtained from BD Biosciences and Cell Signaling Technology, respectively.

### Cell Viability Assay

To measure the cytotoxicity of ISLA, cells were seeded on 96-well culture plates at 5 × 10^3^/well/100 μL, allowed to adhere, and then incubated with or without indicated concentrations of ISLA. After 48 h, 10 μL of Cell Counting Kit-8 (CCK, Dojindo Laboratories, Kumamoto, Japan) solution were added into each well and the absorbance at 540 nm was measured with a SpectraMaxi3 Multi-mode reader (Molecular Devices, Sunnyvale, CA, United States) after incubation for 1 h.

### Preparation of Conditioned Medium (CM)

HT1080 cells were treated with or without indicated concentrations of ISLA in 10% FBS/RPMI1640 media. After 24 h, cells were washed twice with 0.5% FBS/RPMI1640 media and then further incubated for 24 h in 0.5% FBS/RPMI media. Culture media were harvested by centrifugation at 12,000 rpm for 15 min at 4°C and the supernatants were collected as the CM.

### Gelatin Zymography and MMPs Activity Assay

The MMP-2 and MMP-9 activities to degrade gelatin were measured by zymography. In brief, cells were pretreated with indicated concentrations of ISLA in serum-free RPMI for 12 h and then stimulated with PMA (20 nM) for an additional 24 h. Thereafter, culture supernatants were collected and centrifuged at 12,000 rpm for 15 min at 4°C to remove cell debris. The equivalent volumes of culture supernatants were electrophoresed on an 8% sodium dodecyl sulfate-polyacrylamide gel (SDS-PAGE) containing 0.1% gelatin as substrate. After washing with washing buffer (50 mM Tris-HCl, pH 7.5, 100 mM NaCl, 2.5% Triton X-100), gels were incubated in activation buffer (50 mM Tris-HCl, pH 7.5, 150 mM NaCl, 10 mM CaCl_2_, 0.02% NaN_3_, 1 μM ZnCl_2_) at 37°C for 24–48 h. Gels were stained with Coomassie Brilliant Blue R-250 solution (Bio-Rad Laboratories, Hercules, CA, United States) for 30 min and de-stained with 10% isopropanol/10% acetic acid (v/v) solution. The gelatinolytic MMP-9 activities were detected at 92 kDa and MMP-2 were at 72 and 64 kDa as transparent bands against dark blue background. MMPs activity in culture supernatants of ISLA-treated or untreated HT1080 cells was quantitated using MMPs Activity Assay Kit (Cat. No. ab112146, Abcam, Cambridge, MA, United States) according to the manufacturer’s protocol. Green fluorescence intensity was measured using a SpectraMaxi3 Multi-mode reader at Ex/Em 490/525 nm.

### *In vitro* Cell Migration Assays

For Transwell^®^ migration assay, HT1080 cells or HUVECs (1 × 10^4^) suspended in 100 μL serum-free RPMI 1640 media or Endothelial Cell Growth Basal Medium-2 (EBM-2), respectively, were loaded on upper chamber of each Transwell^®^ chamber (10 mm diameter, 8 μm pore size polycarbonate membrane, Corning, Corning, NY, United States). In lower chambers, 600 μL 10% FBS/RPMI1640 media or EGM-2 were added. After incubation in 5% CO_2_ incubator at 37°C, cells remained in upper surface of the membrane were removed by wiping with a cotton swab. Migrated cells in lower surface were stained with 0.2% crystal violet/20% methanol (w/v) solution and then counted under a phase contrast microscope. For scratch migration assay, cells (1 × 10^4^/well/100 μL) cultured on 96-well culture plates to about 90% confluent were pre-treated with 25 μg/mL mitomycin C for 30 min. Using a 96-pin Wound Maker (IncuCyte, Essen BioScience, Ann Arbor, MI, United States), wounds were made on the confluent monolayers according to the manufacturer’s protocol. After plates were installed in the IncuCyte chamber (Essen BioScience), they were incubated with or without ISLA in 5% CO_2_ incubator at 37°C and the wound images were captured every 3 h using an IncuCyte Zoom (Essen BioScience). The relative wound migration was calculated based on the wound width at 0 h.

### *In vitro* Cell Invasion Assays

Transwell^®^ invasion assay and scratch wound invasion assay were performed as a migration assay using Matrigel (diluted to 1:4 with serum-free RPMI) as the intervening invasive barrier. Three-dimensional (3D) invasion assay was performed with the Cultrex 96-well 3D Spheroid Cell Invasion Assay (Trevigen, Gaithersburg, MD, United States) according to the manufacturer’s protocol. In brief, cells (3 × 10^5^) suspended in 50 μL prechilled spheroid formation ECM were added to a Corning 96-well Clear Round Bottom Ultra Low Attachment Microplate (Corning). After centrifugation for 3 min at 200 ×*g*, cells were incubated to assemble into spheroids. After 3 days, prechilled invasion matrix (50 μL) was added onto the each well and plates were incubated for 1 h at 37°C to enhance gel formation. Culture media with or without indicated concentrations of ISLA were added and then the plates were further incubated in 5% CO_2_ incubator at 37°C for 5 days. Invasion of cells into surrounding matrix were observed and photographed every 24 h.

### Western Blot Analysis

Whole cell lysates and nuclear/cytosolic fractions were prepared using M-PER Mammalian Protein Extraction Reagent and NE-PER Nuclear and Cytosolic Extraction Reagent (Thermo Scientific, Rockford, IL, United States), respectively, according to the manufacturer’s instruction. Protein concentrations were determined using a Bicinchoninic Acid (BCA) Kit (Sigma) and then equal protein aliquots (25 μg) were resolved by SDS-PAGE and immunoblotted as described previously using specific antibodies (Kim et al., 2013). The levels of each protein were measured under the ChemiDoc Touch Imaging System (Bio-Rad, Hercules, CA, United States) with the Clarity western ECL substrate (Bio-Rad).

### Proteome Profiler Antibody Array

The levels of angiogenesis-related proteins in the ISLA-treated or untreated HT1080 CMs was evaluated using a Proteome Profiler Human Angiogenesis Array Kit (R&D Systems, Minneapolis, MN, United States) according to the manufacturer’s instruction. Blots were visualized using the ChemiDoc Touch Imaging System and the Clarity western ECL substrate.

### Fluorescence Immunocytochemistry for HIF-1α Nuclear Translocation

Cell grown on 35 mm glass bottom dishes (SPL Lifesciences, Pocheon, Republic of Korea) were treated with indicated concentrations of ISLA for 12 h and then stimulated with CoCl_2_ (200 μM) for 6 h. After being washing three times with cold PBS, the cells were fixed with 4% paraformaldehyde/PBS (v/v) for 30 min at room temperature (RT). Fixed cells were permeabilized and blocked for 30 min at RT with ABS buffer (1 M Tris-Base, 1.5 M NaCl, pH 7.5) containing 0.1% Triton X-100 and 3% goat serum. Localization of HIF-1α was visualized by staining with Alexa Fluor 488-conjugated rabbit anti-HIF-1α antibody (Abcam, diluted 1:500 in ABS buffer) for 3 h at RT. After counterstaining nucleus with DAPI, cells were observed under a fluorescence microscope (Eclipse Ti, Nikon, Tokyo, Japan).

### HUVECs Tube Formation Assay

The ability of HUVECs to form capillary-like tubular structure was measured using a Cultrex *in vitro* angiogenesis assay kit (Trevigen, Gaithersburg, MD, United States). In brief, 50 μL ice-chilled basement membrane extract (BME) was carefully added on a 96-well culture plate and solidified at 37°C for 30 min. HUVECs (5 × 10^4^) pretreated with or without ISLA for 12 h were suspended in 100 μL EGM-2 and then added into each well containing BME. After 4 h, tube formation was visualized through phase contrast inverted microscope.

### Chick Chorioallantoic Membrane (CAM) Assay

Fertilized chicken eggs were obtained from Pulmuone (Seoul, Republic of Korea). We designated this time point as the chick embryonic development (ED) day 0 and eggs were incubated in an egg incubator (R-COM, Gimhae, Republic of Korea) at 37°C with 65% humidity. On ED day 3, albumin was carefully removed using a syringe and then a round window was made on the blunt end with air sac. After covering the windows with adhesive tape, the eggs were returned to the egg incubator. On ED day 6, 5 mm disks loaded with ISLA (100 μg) and/or VEGF (200 ng) were placed on the CAM of individual embryos and further incubated for 3 days. The vasculature was macroscopically observed and photographed.

### Statistics

Data are expressed as the mean ± standard deviation (SD). Statistical significance mean value between two groups was analyzed with Student’s *t*-test. Treatment efficacy was analyzed with one-way ANOVA by Dunnett’s test. All variables were analyzed with GraphPad Prism Software (Version 5.03, GraphPad, San Diego, CA, United States) and a value of *p <* 0.05 was considered to be statistically significant.

## Results

### ISLA Suppressed the PMA-Induced Increase in MMP Activity Without Cytotoxicity

**FIGURE 1:**
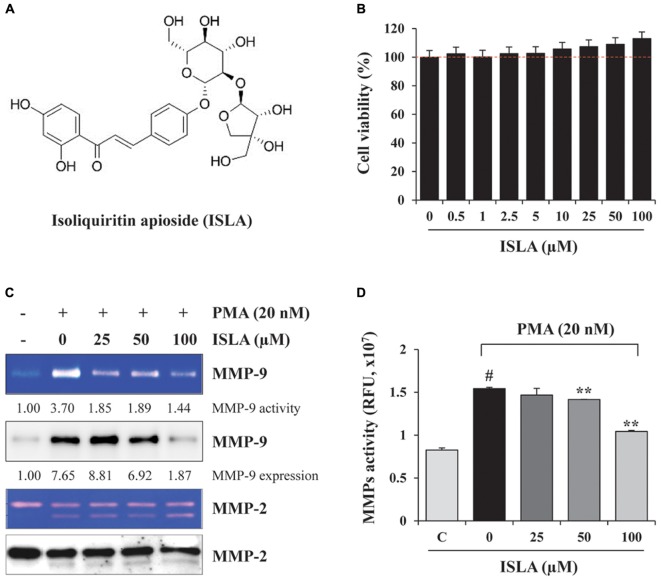
Isoliquiritin apioside (ISLA) at non-cytotoxic doses reduced matrix metalloproteinase (MMP) activity in HT1080 cells. **(A)** The chemical structure of ISLA. **(B)** HT1080 cells were incubated with increasing concentrations of ISLA up to 100 μM for 48 h. Relative cell viability compared with untreated control cells was determined by the CCK-8 assay and expressed as the mean ± SD from three independent experiments performed in triplicate. **(C)** The MMP-9 activity and levels in ISLA-treated and untreated HT1080 conditioned medium (CM) was measured by gelatin zymography and western blotting, respectively. The relative band intensities were calculated using ImageJ software. **(D)** MMP activity in the ISLA-treated and untreated HT1080 CM was measured using the fluorometric green MMP substrate. Fluorescence intensity monitored at an excitation/emission of 490/525 nm was expressed as the mean ± SD (*n* = 3 per group). ^#^*p* < 0.01 vs. untreated control, ^∗∗^*p* < 0.01 vs. ISLA-untreated control.

To evaluate the *in vitro* anti-metastatic potential of ISLA, we first determined the potential cytotoxic effects of ISLA on HT1080 cells using the CCK-8 assay. As shown in Figure 1B, the cell viability of HT1080 cells treated with the indicated concentrations of ISLA for 48 h was not decreased but was slightly increased by approximately 10% with 100 μM ISLA, compared with untreated control cells. HT1080 cells were therefore treated with 100 μM ISLA, the maximum concentration evaluated, in subsequent experiments. Because it is known that MMPs play a major role in promoting metastasis of cancer cells (Deryugina and Quigley, 2006; Halbersztadt et al., 2006; Alaseem et al., 2017), we first examined the proteolytic activity and expression level of MMP-2 and MMP-9 in ISLA-treated and untreated HT1080 CM. Using gelatin zymography, we found that PMA stimulation remarkably increased MMP-9 activity, whereas ISLA treatment efficiently suppressed the PMA-induced gelatinolytic MMP-9 activity in HT1080 cells. In addition, ISLA treatment decreased the PMA-induced increase in MMP-9 production in HT1080 CM. The MMP-2, which is also known to degrade gelatin, was increased into active form by PMA stimulation, however, ISLA did not inhibit the activity of MMP-2 (Figure 1C). Analysis of all MMPs activity in HT1080 CM revealed that ISLA treatment reduced MMPs activity of HT1080 cells in a dose dependent manner (*F* = 91.44, *p* < 0.0001, one-way ANOVA) (Figure 1D).

### ISLA Suppressed the *in vitro* Migration and Invasion Ability of HT1080 Cells

**FIGURE 2:**
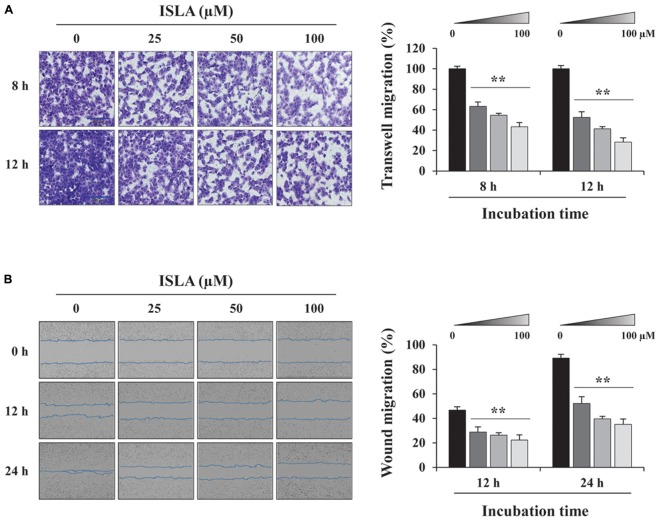
Isoliquiritin apioside suppressed the migration of HT1080 cells. **(A)** HT1080 cells were pretreated with or without ISLA for 12 h and then subjected to migration across a Transwell^®^ membrane. After 8 and 12 h, the number of migrated cells in five random fields was counted using phase contrast inverted microscopy after staining with Crystal Violet solution. Relative migration compared with untreated control cells was calculated and expressed as the mean ± SD (*n* = 5). Data are representative of three independent experiments. **(B)** After inflicting injury wounds in HT1080 cell layers using a 96-pin wound maker, cells were treated with or without ISLA and then monitored every 3 h using an IncuCyte Zoom. The relative wound migration in each group at 12 and 24 h was calculated based on the wound width at 0 h using ImageJ software. Data are expressed as the mean ± SD obtained from triplicate samples. ^∗∗^*p* < 0.01 vs. ISLA-untreated control.

**FIGURE 3:**
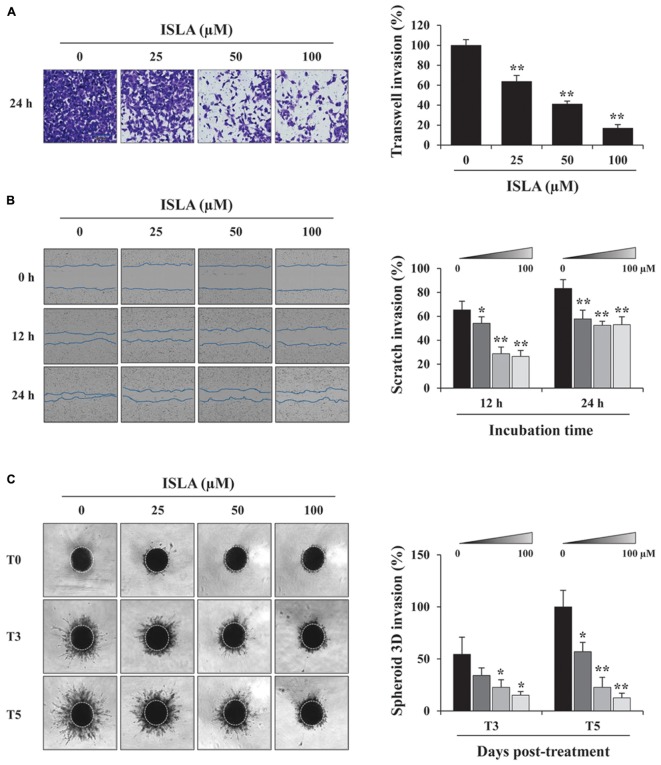
Isoliquiritin apioside suppressed the invasion of HT1080 cells. **(A)** HT1080 cells were pretreated with or without ISLA for 12 h and allowed to invade across a Matrigel-coated Transwell^®^ membrane. After 24 h, the number of invaded cells was counted using phase contrast inverted microscopy after staining with Crystal Violet solution. Relative invasion compared with untreated control cells was calculated and expressed as the mean ± SD from five random fields. Data are representative of three independent experiments. **(B)** A scratch-wound was made on HT1080 cell layers using a 96-pin wound maker. ISLA-containing Matrigel was added to the cells and allowed to polymerize for 30 min at 37°C. Cells were incubated in ISLA-containing medium in a 5% CO_2_ incubator at 37°C and monitored every 3 h using an IncuCyte Zoom. The relative wound invasion in each group at 12 and 24 h was calculated based on the wound width at 0 h, using ImageJ software, and expressed as the mean ± SD from triplicate experiments. **(C)** Cells assembled to compact spheroids were incubated in the presence or absence of ISLA for 5 days. The images of stellate-like spheroids were photographed at 3 and 5 days post-treatment (T3 and T5), and the reactive spheroid area was determined using ImageJ software. The data are expressed as means ± SD from triplicate samples. ^∗^*p* < 0.05, ^∗∗^*p* < 0.01 vs. ISLA-untreated control.

To confirm the inhibitory effect of ISLA on the migration of HT1080 cells, we first examined the ability of ISLA-treated or untreated cells to migrate across a Transwell^®^ membrane. As shown in Figure 2A, ISLA treatment dramatically inhibited serum-induced migration in a dose-dependent manner, showing reductions of 56.8 and 71.8% at 100 μM compared with the control cells at 8 and 12 h, respectively (8 h; *F* = 259.7, *p* < 0.0001, 12 h; *F* = 312.0, *p* < 0.0001, one-way ANOVA). In scratch-wound migration assays, untreated control HT1080 cells migrated across the scratch-wound region, leading to 46.7 and 89.1% closure of the wound at 12 and 24 h, respectively. However, 25, 50, and 100 μM ISLA treatment significantly inhibited wound migration at 24 h by 41.5, 55.6, and 60.6%, respectively, compared with that of control cells (12 h; *F* = 22.01, *p* < 0.0001, 24 h; *F* = 87.3, *p* < 0.0001, one-way ANOVA) (Figure 2B). We next examined the ability of ISLA-treated or untreated HT1080 cells to invade across a Matrigel barrier. In the Transwell^®^ invasion assay, ISLA treatment remarkably suppressed serum-induced invasion in a dose-dependent manner, leading to approximately 83.2% inhibition with 100 μM ISLA compared with untreated control cells (*F* = 272.5, *p* < 0.0001, one-way ANOVA) (Figure 3A). Untreated control HT1080 cells reduced the Matrigel-coated scratch-wound by 65.4 and 83.3% at 12 and 24 h, respectively, while 100 μM ISLA inhibited invasion by approximately 40 and 63.6% of that of control cells at 12 and 24 h, respectively (12 h; *F* = 52.72, *p* < 0.0001, 24 h; *F* = 26.28, *p* < 0.0001, one-way ANOVA) (Figure 3B). In addition, invasion into the surrounding ECM from 3D spheroids was also dramatically inhibited by 100 μM ISLA in a dose-dependent manner, compared with that of control cells, showing reductions of 72.1 and 87.3% at 3- and 5-days post-treatment, respectively (T3; *F* = 9.006, *p* = 0.0061, T5; *F* = 42.39, *p* < 0.0001, one-way ANOVA) (Figure 3C).

### ISLA Blocked PMA-Induced MAPK Phosphorylation as Well as NF-κB Activation in HT1080 Cells

**FIGURE 4:**
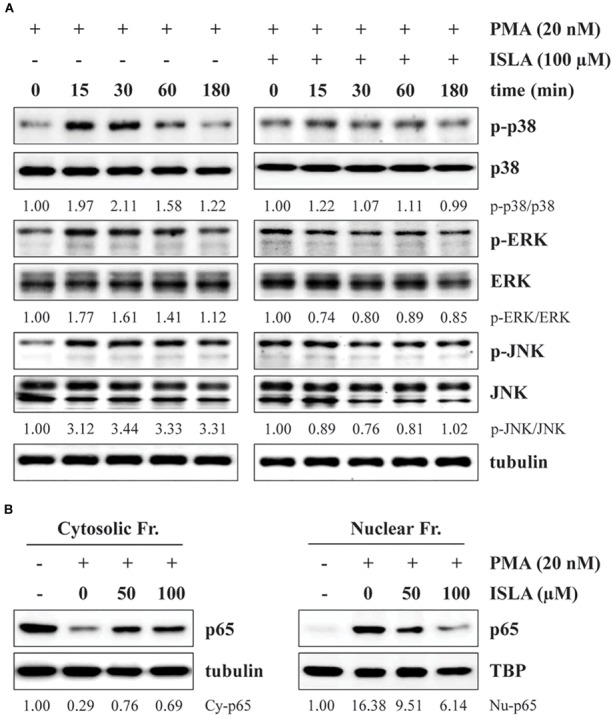
Isoliquiritin apioside inhibits PMA-induced MAPK and NF-κB activation in HT1080 cells. **(A)** Cells were pretreated with or without 100 μM ISLA for 12 h and then stimulated with 20 nM PMA for the indicated times. Total cell lysates were prepared and evaluated for MAPK activation using western blotting. The band intensities of phosphorylated p38, ERK, and JNK relative to the respective total protein levels were calculated using ImageJ software after normalization to tubulin expression. **(B)** Cells were pretreated with or without 50 and 100 μM ISLA for 12 h and then stimulated with 20 nM PMA for 30 min. Cytosolic and nuclear fractions were isolated, and the levels of the NF-κB p65 subunit were determined by western blotting. The relative intensities of the NF-κB p65 subunit in cytosolic and nuclear fractions were calculated using ImageJ software after normalization to tubulin and TBP expression, respectively. Data are representative of three independent experiments.

It has been reported that activation of NF-κB and MAPKs, including p38, ERK, and JNK, is involved in the PMA-induced increases in MMP activity and expression (Yan and Boyd, 2007). Thus, we next examined whether ISLA downregulates these signaling pathways. As previously reported, PMA stimulation rapidly increased the phosphorylation of p38, ERK, and JNK in HT1080 cells (Kim et al., 2013), while in ISLA-treated HT1080 cells, PMA-induced p38 phosphorylation was partially inhibited, and phosphorylation of ERK and JNK was almost completely blocked (Figure 4A). Activation of the transcription factor NF-κB requires phosphorylation and degradation of IκBα, followed by translocation of the p65 subunit of NF-κB from the cytosol to the nucleus, where it binds to the promoter regions of target genes (Sarkar et al., 2008). As shown in Figure 4B, in untreated control cells, PMA stimulation significantly increased the nuclear protein level of the p65 subunit, whereas ISLA treatment efficiently decreased the nuclear/cytosolic ratio of the p65 subunit in a dose-dependent manner. Together, these data indicated that ISLA inhibited the metastatic potential of HT1080 cells, without inducing cytotoxicity, by reducing MMP activity via suppression of MAPK and NF-κB activation.

### ISLA Decreased Production of Angiogenesis-Related Proteins and Suppressed the HIF-1α Pathway in HT1080 Cells Under Hypoxic Conditions

**FIGURE 5:**
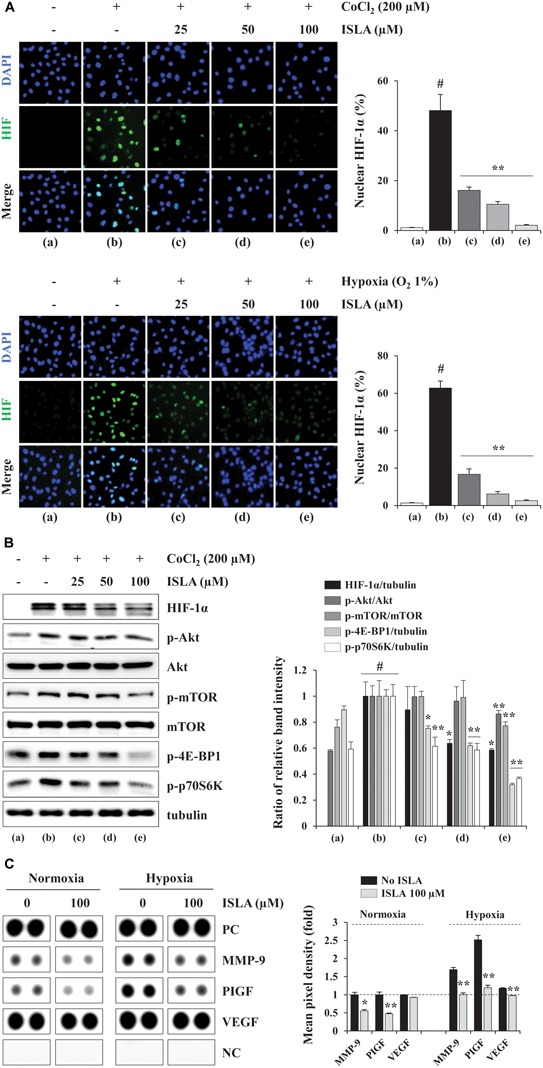
Isoliquiritin apioside suppressed HIF-1α nuclear expression and reduced the production of pro-angiogenic proteins of HT1080 cells. **(A)** Cells grown in glass bottom dishes were treated with the indicated concentrations of ISLA for 12 h and then exposed to hypoxic conditions including CoCl_2_ stimulation and 1% O_2_ (*for 6 h. After staining with Alexa Fluor 488-HIF-1α antibody and counterstaining with DAPI, nuclear HIF-1α levels were measured. Data are representative of two independent experiments and expressed as the means ± SD of five selected fields per sample. **(B)** Cells were treated with or without ISLA for 12 h and then stimulated with 200 μM CoCl_2_ for 6 h. The levels of HIF-1α as well as molecules downstream of HIF-1α in total cell lysates were detected by western blotting. The relative band intensities were calculated using ImageJ software after normalization to tubulin expression and expressed as the mean ± SD from two independent experiments. **(C)** The levels of angiogenesis-related proteins were measured in HT1080 CMs obtained from ISLA-treated or untreated cells incubated under normoxic (20% O_2_) and hypoxic (1% O_2_) conditions. The pixel intensities from duplicate dots were measured, and the relative values compared with untreated control cells under normoxia were calculated. The data are representative of two independent experiments and expressed as the means ± SD of duplicate dots. ^#^*p* < 0.01 vs. untreated control, ^∗^*p* < 0.05, ^∗∗^*p* < 0.01 vs. ISLA-untreated control.*)

Hypoxia in the tumor microenvironment (TME) is critical for the formation of blood vessels, which promote tumor cell survival, metastasis to distant organs, and resistance to cell death (Finger and Giaccia, 2010). Under hypoxic conditions, the key transcription factor, HIF-1α, accumulates in the nucleus, leading to expression of its downstream targets including pro-angiogenic factors (Semenza, 2000, 2010a). To investigate the effect of ISLA on the production of pro-angiogenic factors in cancer cells, we first measured the nuclear accumulation of HIF-1α in response to hypoxic stimuli, including CoCl_2_ stimulation and a low oxygen (1% O_2_) concentration, by fluorescence immunocytochemistry in ISLA-treated or untreated HT1080 cells. As shown in Figure 5A, in untreated control cells, nuclear accumulation of HIF-1α was increased under hypoxic conditions, whereas ISLA treatment significantly decreased the proportion of cells with nuclear HIF-1α in a dose-dependent manner, showing reduction to approximately 5% of control cells at 100 μM ISLA (CoCl_2_; *F* = 184.3, *p* < 0.0001, 1% O_2_; *F* = 646.4, *p* < 0.0001, one-way ANOVA). Using western blotting, we confirmed that ISLA efficiently inhibited hypoxia-induced HIF-1α accumulation and phosphorylation of Akt, mTOR, 4E-BP1, and p70S6K in a dose-dependent manner (Figure 5B). Next, the effect of ISLA on the production of pro-angiogenic factors under normoxic and hypoxic conditions was analyzed in HT1080 CM obtained from ISLA-treated or untreated HT1080 cells. Figure 5C shows that the levels of pro-angiogenic factors such as MMP-9 and placental growth factor (PlGF) were significantly decreased by ISLA treatment under normoxic conditions. Under hypoxic conditions, the levels of MMP-9 and PlGF were increased by 1.69- and 2.52-fold, respectively, compared with those under normoxia conditions, and ISLA also reduced these levels. The levels of VEGF under normoxic and hypoxic conditions were slightly decreased by ISLA treatment. These data indicated that ISLA suppressed the angiogenic potential of HT1080 cells by reducing production of pro-angiogenic factors via suppression of HIF-1α signaling pathway.

### ISLA Suppressed Angiogenic Activities of HUVECs

**FIGURE 6:**
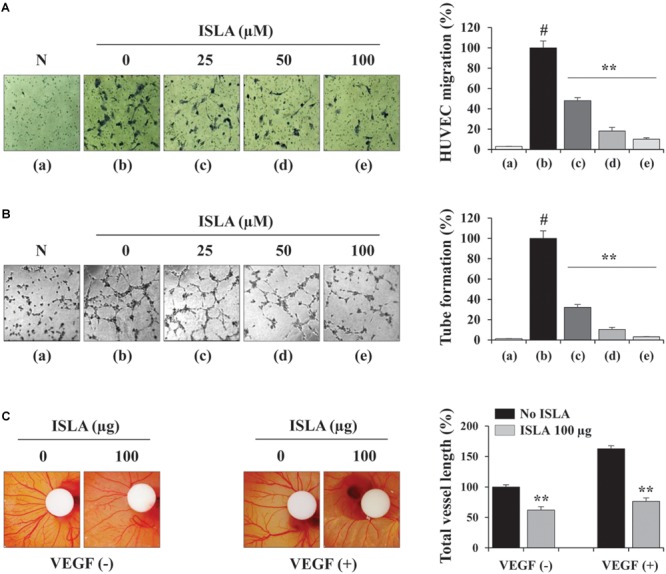
Isoliquiritin apioside decreased the angiogenic abilities of HUVECs. **(A)** HUVECs were treated with or without ISLA for 12 h and suspended in EBM-2. The migration of HUVECs induced by EGM-2 across the Transwell^®^ membrane was evaluated after 15 h. Migration induced by EBM-2 was used as a negative control (N). Relative migration compared with untreated control cells was calculated and expressed as the mean ± SD (*n* = 5). **(B)** HUVECs treated with or without ISLA for 12 h were suspended in EGM-2 and then loaded onto BME-coated wells. After incubating for 4 h, capillary-like tube formation was visualized by phase contrast inverted microscopy. Untreated HUVECs suspended in EBM-2 were used as a negative control (N). The number of tubes was counted in triplicate samples, and relative tube formation compared with untreated HUVECs was calculated. Data are expressed as means ± SD (*n* = 3). **(C)** On ED day 6, filter disks containing ISLA (100 μg) and/or VEGF (200 ng) in PBS (20 μL) were carefully loaded onto the CAM. After sealing the window with adhesive tape, the eggs were incubated for 3 days in an egg incubator. On ED day 9, the vessels in the eggs were photographed, and the total vessel length was measured by ImageJ software in three samples per group. Relative vessel length compared with HUVECs without ISLA and VEGF treatments was calculated and expressed as the mean ± SD (*n* = 3). ^#^*p* < 0.01 vs. untreated control, ^∗∗^*p* < 0.01 vs. ISLA-untreated control.

Angiogenesis, which involves the formation of new blood vessels, is an essential step in tumor progression and metastasis (Rak et al., 1995). It is controlled by numerous growth factors released by cancer cells, which promote EC proliferation, migration, adhesion, and the formation of tubular structures (Coultas et al., 2005). To determine the direct effects of ISLA on ECs, we first examined the ability of ISLA-treated or untreated HUVECs during EGM-2-induced migration across a Transwell^®^ membrane. Figure 6A shows that untreated control HUVECs efficiently migrated toward EGM-2 but not EBM-2, whereas ISLA-treated HUVECs showed reductions of 51.9, 81.9, and 89.9% after treatment with 25, 50, and 100 μM ISLA, respectively, compared with the migration of control HUVECs (*F* = 471.4, *p* < 0.0001, one-way ANOVA). Untreated control HUVECs showed complete EGM-2-induced tubular structures, while ISLA-treated HUVECs showed weak EGM-2-induced tubular structures in a dose-dependent manner (*F* = 577.1, *p* < 0.0001, one-way ANOVA) (Figure 6B). Using the CAM assay, we observed spontaneous angiogenesis on ED day 6, and the topical application of ISLA suppressed angiogenesis by approximately 37% compared with that of vehicle alone on ED day 9. Furthermore, VEGF stimulation strongly induced angiogenesis in terms of sprouting, length, and thickness of vessels, whereas ISLA efficiently blocked VEGF-induced angiogenesis to approximately 46.8% of the vehicle control level (Figure 6C), indicating that ISLA directly suppressed the angiogenic response of ECs.

## Discussion

Carcinogenesis consists of multistage process that begins with irreversible cellular transformation, expands by aberrant proliferation, and acquires invasive and angiogenic potential, consequently leading to metastatic growth. For the several decades, development of anti-cancer drugs has focused on the cytostatic and cytotoxic potency. For these cytotoxic anti-cancer agents, there are some limitations because they can also damage to rapidly dividing normal cells such as bone marrow, digestive tract, and hair follicles. In addition, they have little effect on the malignant properties such as invasion, metastasis, and angiogenesis (Thambi and Bharat, 2004). Therefore, non-toxic anti-metastatic and anti-angiogenic agents which can restrain the spread of malignant cancer cells are getting attention as anti-cancer drugs, (Gao et al., 2014).

Numerous recent studies have reported that tumor cells closely interact with the surrounding ECM and neighboring normal stromal cells, which constitute the TME (Tredan et al., 2007; Whiteside, 2008; Mbeunkui and Johann, 2009). This interaction between tumor cells and the TME can influence the process of carcinogenesis and contribute to determining the hallmarks of cancer. In contact-dependent mechanisms, cell–cell and cell–ECM adhesion plays an essential role. In contact-independent mechanisms, soluble factors secreted from cancer cells, including growth factors, chemokines, and cytokines, efficiently affect stromal cells, ECs, and immune cells in the TME. In addition, cells in the TME also provide essential signals for tumor progression, suggesting that targeting the TME is an effective therapeutic strategy in cancer therapy (Cairns et al., 2006; Danhier et al., 2010; Wood et al., 2014). Over the last decade, great efforts have been made to identify and develop therapeutic agents interfering with tumor cell–TME interactions or inhibiting the pathways activated by TME. Agents at advanced preclinical or clinical stages of development that primarily target TME have been involved in four major strategies: targeting the tumor vasculature, hypoxia in the TME, cancer-induced inflammation, and communication between tumor cells and TME (Joyce, 2005; Cairns et al., 2006).

The vascularization in tumors is tightly regulated by pro- and anti-angiogenic factors released from both tumor cells and surrounding stromal cells through autocrine and paracrine pathways. Overgrown tumor cells are in a state of oxygen deficiency (hypoxia), which leads to an angiogenic switch for secretion of pro-angiogenic factors, including VEGF, FGF, PDGF, and EGF (Choi et al., 2003). Hypoxia causes the sprouting of new blood vessels via facilitating the proliferation, migration, and organization of ECs in capillary structures, which in turn increases the survival, metastasis, and EMT of cancer cells and the resistance against anti-cancer therapies (Pouyssegur et al., 2006). In these processes, overexpression and stabilization of HIF-1α and activation of PI3K/AKT/mTOR, MAPK, and NF-κB pathways are involved (Semenza, 2010b; Karar and Maity, 2011). Agents targeting HIF-1α signaling such as EZN-2968 (HIF-1α antisense mRNA) and PX-478 (small molecule inhibitor of HIF-1α) have been effective treatments for advanced solid tumors and lymphoma in phase I clinical trials (Jeong et al., 2014; Welsh et al., 2004). In addition, several phytochemicals, such as curcumin, wogonin, and resveratrol, and herbal medicines, such as an ethanol extract of baked *Gardeniae Fructus* and an ethanol extract of *Annona atemoya*, have been reported to have anti-angiogenic effects through inhibition of the HIF-1α pathway (Song et al., 2013; Yi et al., 2014; Bae et al., 2006; Im et al., 2016).

*Glycyrrhizae radix rhizome*, the root of *G. uralensis*, is a perennial plant of the Leguminosae family. GR is sweet in flavor and neutral in nature, so it is primarily used as a harmonizer in Chinese herbal formulas. In pharmacological studies, GR has been shown to prevent ulcers, relieve gastrointestinal smooth muscle spasms, promote pancreatic juices, reduce cough, protect the liver, and induce antibacterial, antiviral, anti-inflammatory, and anti-allergic effects (Lee et al., 2013; Kim et al., 2013; Jeon et al., 2016). Several active components have been isolated from GR, including glycyrrhizin, liquiritin, liquiritigenin, isoliquiritin apioside, liquiritin apioside, and isoliquiritigenin. Among them, glycyrrhizin, a glycoconjugated triterpene, was the first identified to have antiviral activity and has been used to treat patients with chronic hepatitis B and C due to its potent anti-inflammatory activity (Mbeunkui and Johann, 2009). Glycyrrhizin attenuated high-fat diet-induced obesity in Wistar rats by increasing insulin receptor expression and activating NrF2 and the homooxygenase-1 pathway (Abo El-Magd et al., 2018). In addition, glycyrrhizin has been reported to induce apoptosis in various cancers, including breast, liver, lung, and pancreatic cancers, by the generation of reactive oxygen species, cell cycle arrest, and/or autophagy (Huang et al., 2014). Furthermore, combined treatment with glycyrrhizin and cisplatin in the PDX mouse model efficiently suppressed the growth of lung tumor masses with no severe side effects (Wu et al., 2018). In mice inoculated with the B16 melanoma, glycyrrhizin treatment efficiently inhibited pulmonary metastasis through regulation of tumor-associated Th2 cells (Kobayashi et al., 2002). In our experiments, we observed that glycyrrhizin induced cell death of HT1080 cells at concentrations > 25 μg/mL and efficiently inhibited PMA-induced MMP-9 activity at non-toxic concentrations. As reported in previous studies, liquiritin, liquiritigenin, and isoliquiritigenin decreased the cell viability of HT1080 cells, and weakly suppressed PMA-induced MMP-9 activity (data not shown). While various pharmacological effects of active ingredients in GR, including anti-cancer activities such as induction of cancer cell death and inhibition of metastasis, are well known, there are not many reports of efficacy studies on ISLA. In an *in vivo* experimental muscle cramp model in the rat gastrocnemius muscle, ISLA injection at 20 μmol/kg consistently inhibited electrically induced tetanic contractions during the latter phase of contraction (Lee et al., 2013). In addition, ISLA exhibited marked inhibitory activity against H_2_O_2_ and 4NQO-induced DNA damage confirmed by SOS chromotest and Comet assay (Kaur et al., 2009). The authors of this paper mentioned the possibility of the cancer prevention of ISLA in that reactive oxygen species play a pivotal role on the tumor initiation through oxidative damage of DNA. However, the anti-cancer effects of ISLA on cancer cells and ECs have not been previously reported. This study is therefore the first study to describe the anti-metastatic and anti-angiogenic activities of ISLA and to elucidate its underlying mechanisms.

**FIGURE 7:**
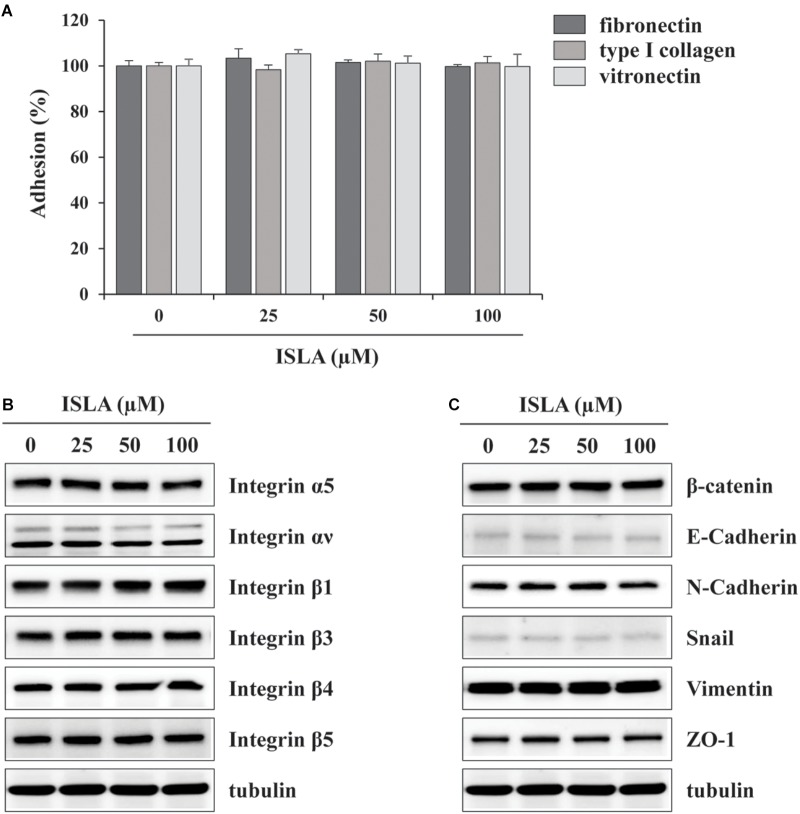
Isoliquiritin apioside does not affect the adhesion ability and expression of EMT-related proteins and integrins of HT1080 cells. **(A)** The wells of 96-well culture plates were coated overnight at room temperature with 5 μg/mL fibronectin (Sigma), 0.3% type I collagen solution (Cellmatrix type I-A; Nittazerachin Co., Osaka, Japan), and 10 μg/mL vitronectin (Sigma) in a volume of 50 μL. After blocking with 200 μL RPMI containing 3% BSA, ISLA-treated or -untreated HT1080 cells suspended in serum-free RPMI (1 × 10^5^/200 μL) were added to ECM-coated wells and then allowed to adhere for 1 h at 37°C. Unbound cells were washed with PBS three times, and the attached cells were fixed and stained with 0.2% crystal violet/20% methanol (w/v) solution. After washing with distilled water, dye was lysed with 1% SDS and measured absorbance at 560 nm using SpectraMaxi3 Multi-mode reader. Data are expressed as means ± SD (*n* = 3). **(B,C)** Cells were treated with or without ISLA for 24 h and the whole cell lysates were extracted. The levels of EMT-related proteins and integrins were measured by western blotting using EMT Antibody Sampler Kit (Cell Signaling Technology, cat. no. 9782) and Integrin Antibody Sampler Kit (Cell Signaling Technology, cat. no. 4749).

In the present study, we demonstrated that ISLA efficiently suppressed the metastatic migration and invasion of HT1080 cells confirmed by Transwell^®^ migration/invasion assay, scratch-wound migration/invasion assay, and 3D spheroid invasion. Interestingly, at effective doses up to 100 μM, cell proliferation was not affected, but rather slightly increased in a dose-dependent manner (Figures 1–3), indicating that ISLA is a safe and non-toxic anti-metastatic component which does not affect cancer cell viability and induce cancer cell death. Adhesion of HT1080 cells to ECMs including fibronectin, type I collagen, and vitronectin was not affected by ISLA treatment. Levels of integrins in ISLA-treated HT1080 cells were similar to those of ISLA-untreated HT1080 cells, indicating that ISLA has no inhibitory effect on the adherence between cancer cells and ECMs. In addition, epithelial-mesenchymal transition (EMT)-related proteins, including β-catenin, E-cadherin, N-cadherin, snail, vimentin, and ZO-1, by which cause the loss of cell–cell junction and dissemination of cancer cells, were not regulated by ISLA treatment (Figure 7). In studies examining the mechanism of anti-metastatic action, we observed that ISLA treatment almost completely blocked PMA-induced MAPK activation and inhibited nuclear translocation of the NF-κB p65 subunit, resulting in decreased MMP activity, which is crucial for cancer metastasis (Figures 1, 4). In addition, ISLA reduced the levels of pro-angiogenic factors such as MMP-9, PlGF, and VEGF in HT1080 CM via suppression of the HIF-1α/Akt/mTOR pathway, supporting the anti-angiogenic activity of ISLA in cancer cells (Figure 5). Moreover, ISLA inhibited the ability of HUVECs to migrate across a Transwell^®^ membrane and establish a tubular structure and significantly reduced vessel formation in the CAM assay, indicating that ISLA directly regulated tumor cells as well as ECs to limit tumor progression. Blood vessels provide tumor cells with sufficient oxygen and nutrients and remove toxic waste products, thus solid tumor cannot grow to measurable size without developing functional vascular networks. In addition, tumor cells intravasate into circulation and extravasate from circulation via these vessels, leading to the establishment of secondary metastatic foci (Rak et al., 1995; Folkman, 2002). In this study, we demonstrated that ISLA remarkably suppressed migration, invasion, and angiogenesis of malignant cancer cells as well as ECs.

To improve the efficiency and to lower the cost for cancer care, many researchers are trying to find a more effective and safe agents. In this regard, cancer treatment with natural phytochemical compounds is an emerging strategy to delay or cure cancers. Certain bioactive components, including curcumin from turmeric, resveratrol from graphs, genistein from soybean, and tea polyphenols from green tea have been demonstrated for their anti-cancer activities. In the United States, approximately 50–60% of cancer patients utilize these agents as complementary and alternative medicine (Wang et al., 2012).

Collectively, these results strongly suggest that ISLA can be developed as a potent and safe anti-cancer drug for treating patients with highly metastatic malignant cancers. For application in the clinical studies and further in chemoprevention and treatment of cancer patients, bioavailability and toxicity of ISLA are considered. Therefore, to verify *in vivo* anti-cancer efficacy of ISLA, we are currently investigating whether ISLA is able to suppress the metastasis of malignant cancer cells in experimental animal model. In addition, assessment of *in vivo* safety of ISLA is also in progress.

## Author Contributions

AK and JM conceived and designed the experiments. AK performed the experiments, analyzed the data, and wrote the manuscript. All authors reviewed the results and approved the final version of the manuscript.

## Conflict of Interest Statement

The authors declare that the research was conducted in the absence of any commercial or financial relationships that could be construed as a potential conflict of interest.
